# Adopted daughters and adopted daughters-in-law in Taiwan: a mortality analysis

**DOI:** 10.1098/rsos.171745

**Published:** 2018-03-21

**Authors:** Siobhán M. Mattison, Edmond Seabright, Adam Z. Reynolds, Jingzhe (Bill) Cao, Melissa J. Brown, Marcus W. Feldman

**Affiliations:** 1 Department of Anthropology, University of New Mexico, Albuquerque, NM, USA; 2 Department of Biology, Boston University, Boston, MA, USA; 3 Harvard-Yenching Institute, Cambridge, MA, USA; 4 Department of Biology, Stanford University, Stanford, CA, USA

**Keywords:** fictive kinship, minor marriage, evolutionary demography, alloparenting

## Abstract

Adoption is sometimes considered paradoxical from an evolutionary perspective because the costs spent supporting an adopted child would be better spent on rearing one's own. Kin selection theory is commonly used to solve this paradox, because the adoption of closely related kin contributes to the inclusive fitness of the adoptive parent. In this paper, we perform a novel test of kin selection theory in the context of adoption by asking whether adopted daughters-in-law, who contribute directly (i.e. genealogically) to the perpetuation of their adoptive families' lineages, experience lower mortality than daughters adopted for other purposes in historical Taiwan. We show that both classes of adopted daughter suffer lower mortality than biological daughters, but that the protective effect of adoption is stronger among daughters who were not adopted with the intention of perpetuating the family lineage. We speculate as to the possible benefits of such a pattern and emphasize the need to move beyond typological definitions of adoption to understand the specific costs and benefits involved in different forms of caring for others' children.

## 1. Introduction

Adoption—the transferral of offspring from biological to non-biological parents [1]—arises under numerous circumstances, and differences among these circumstances are likely to affect the degree of alloparental investment in and subsequent welfare of adopted children [2]. Kin adoption has been posited to be a major form of adoption in human societies [3–5], but cases of non-kin adoption have been described in both humans and non-human animals [1,6], suggesting deep evolutionary roots to these practices [3,7]. Indeed, there are many possible reasons for adoption to occur, including the loss of a parent [8,9], the need to balance household composition against resource availability, and the opportunity to create or reinforce alliances among families [10]. Although the variability in adoption is widely recognized from an evolutionary perspective [1,3,7,11], studies of adoptive behaviour commonly operationalize adoption as a binary, static, yes-or-no state. In this paper, we compare two different historical forms of Taiwanese adoption to discern whether functional distinctions between adoptive categories lead to measurably different mortality patterns in children adopted for different purposes.

We use a unique dataset to explore whether specific forms of adoption of unrelated individuals have different consequences vis-à-vis mortality. Our data were produced during Japanese colonial rule of Taiwan between 1895 and 1945—a time and place when adoption was both prevalent and highly varied in terms of household demography and adoptive parental intent. For example, families lacking sons to perpetuate their lineage and provide old-age support could adopt a boy or bring in a son-in-law to serve this purpose. By contrast, girls, among whom adoption was far more common, were most frequently adopted in one of two ways: (i) as *sim-pua*, or adopted daughters-in-law (ADIL) for the purposes of minor marriage or (ii) as adopted daughters (AD) for one of several purposes. In the first form of adoption, parents adopted a girl at a young age to serve as the eventual bride for their co-resident son and to perpetuate the family's biological lineage. In the second form, daughters were more often adopted as stand-ins for biological children, either because the adoptive mother was childless or in the belief that the adoption of a daughter would herald the birth of a biological son (so-called therapeutic adoption; [12–15]). AD, like biological daughters, could provide old-age support (including through prostitution), and might also bring in a son-in-law through uxorilocal marriage [12–15].

Evolutionary theory provides a rationale for why these different forms of adoption might have different effects on mortality. First, kin selection theory [16], which is perhaps the most common explanation for adoptive behaviour, anticipates, all else being equal, greater care or less neglect of individuals who contribute relatively more to an actor's inclusive fitness. Significant evidence comparing biological to non-biological children bolsters this expectation (e.g. [17–19]). However, in a previous comparison of adopted daughters to biological daughters in Taiwan, Mattison *et al*. [20] demonstrated that the mortality of adopted daughters on the whole (i.e. including both ADIL and AD) was lower than that of biological daughters, contradicting a kin-selection hypothesis. They salvaged the kin selection hypothesis in speculating that, within adopted girls, ADIL might experience higher survivorship than AD because ADIL adoption was associated with adoptive parents' desire to perpetuate their biological lineage. In other words, because ADIL represented an investment by adoptive parents in those parents' own inclusive fitness, Mattison *et al*. hypothesized that mortality rates would be lower among ADIL than among AD. In investigating this hypothesis here, we expand on work that uses kin selection to compare the fates of biological and non-biological children (e.g. [20–22]) by analysing, *within* the category of adopted girls, whether specific types of adoption are associated with differences in mortality.

Evolutionary theory also recognizes a number of other possible benefits—apart from genealogical relatedness—to non-biological parents that could contribute to the adoption and welfare of adopted girls [3,6,11,20,23–27]. Indeed, parents should benefit from adoption if the costs of adopting are exceeded by its benefits [28,29]. This context could arise through a number of different mechanisms, including via contributions of adopted children to household economics (e.g. through help in domestic chores) (e.g. [30]), to adoptive parental fitness (e.g. if adopted children act as ‘helpers-at-the-nest’ [31,32]), or through beneficial effects of creating or maintaining alliances with other families [3,23,24]. In the case of Taiwanese AD, ethnographic evidence suggests AD were adopted to remedy perceived demographic deficiencies of the adopting household. These motivations could be associated with a number of possible benefits as described above, as well as with lower costs if the additional child did not overtax the adoptive family economy. Lacking data that allow direct exploration of such broad costs and benefits, we focus here on the narrower benefits posited to accrue to adoptive parents due to genealogical relatedness through ADIL. This hypothesis, as summarized below, anticipates lower mortality in ADIL compared with AD and lower mortality among AD compared with biological daughters.

*Summary of Hypotheses*

H0: There is no difference between ADIL and AD in mortality. The null model allows these two adoption types to be combined and suggests the main contrast should be between biological and non-biological children, not between categories of adopted children.

H1: There is a difference between ADIL and AD in mortality. If this difference is due to kin selection, ADIL should have higher survivorship than AD because ADIL perpetuate the adoptive family lineage.

## 2. Material and methods

The data used in this study were digitized from household registers collected by the Japanese colonial administration between 1895 and 1945 [33]. The administration was assiduous in collecting accurate demographic data [14,34–36]. A single register was kept for each household, which a senior household member was required to update at his/her local police station for all major demographic changes, including births, deaths, marriages, adoptions and migrations. When a new household was formed, all relevant information from the previous register was transcribed to a new register and cross-referenced. Households were visited periodically by police officers—at least twice a year [14]—who would check family composition against the current version of the register. Punishments for failing to report an event were potentially severe [35,37,38], and applied not only to the head of household where the omission occurred, but also to the appointed head of a community of neighbouring families. This collective responsibility ensured extremely high rates of reporting.

### 2.1. Sample selection

Our final sample for analyses of mortality included 31 066 women (of whom 1897 were adopted; see electronic supplementary material) after applying several exclusion criteria. All were born after 1905 and their births were recorded in the database (i.e. they did not enter the database via immigration). Additionally, the identity of the adoptive parents had to be known in order to identify the presence of adoptive siblings in the database, so girls with unknown adoptive parents were also excluded. Girls were further excluded if they had been adopted multiple times, adopted after the age of 10, or adopted into their household of birth, all of which were atypical forms of adoption. Finally, adoptions by a close relative (biological parents, aunts, uncles, cousins or grandparents) were removed from the analysis, because close-relative adoption is extremely rare in our data, comprising only 423 cases before exclusions (1.51% of 21 522 adopted and 0.30% of 140 324 total children, including boys). Electronic supplementary material, figure S1 shows frequencies of close-relative adoptions by receiving kin.

### 2.2. A rule for determining type of adoption

The registers do not explicitly label daughter-in-law (DIL) adoption differently from other forms of adoption. Wolf and associates [14,15] argued *based on ethnographic observation* that parents with a son who adopted a girl were doing so with the likely goal of providing a bride for their son. Rather than assuming this connection *a priori*, we developed an algorithm to identify putative DIL adoptions as follows. We ran a logistic regression on all adopted daughters *with known marital outcomes.* The outcome variable, whether the adopted daughter married her adoptive brother, was coded as 1 if such a marriage was observed and 0 otherwise. We then identified the set of a girl's characteristics that best predicted whether she would subsequently marry her adoptive brother using AIC comparisons. The model confirmed Arthur Wolf's ethnographic insights (table 1), so we used the following rule to identify a DIL adoption: *if a girl's adoptive parents had a biological son alive at the time of adoption who was at most 15 years older than the girl, she was adopted as a DIL; other cases were coded as AD*.

**Table 1.** Predictors of ADIL status for adopted girls with known marital outcomes.^a^

	estimate	s.e.	Pr (>|z|)
(intercept)	110.096	10.803	<0.001***
birth year	−0.059	0.006	<0.001***
age at adoption	−0.131	0.022	<0.001***
number of biological siblings	0.033	0.043	0.441
adoption cancelled^b^	−0.681	0.535	0.203
adoptive brother^c^	4.707	0.151	<0.001***
adoptive brother × adoption cancelled^b,c^	−3.025	0.585	<0.001***
bound feet	−0.380	0.104	<0.001***
birth HH occupation: craftsman^d^	0.671	0.456	0.141
birth HH occupation: labourer^d^	−0.122	0.217	0.574
birth HH occupation: landlord^d^	0.826	0.897	0.357
birth HH occupation: merchant^d^	−0.341	0.230	0.137
birth HH occupation: unknown^d^	−0.493	0.247	0.046*
adopted HH occupation: craftsman^d^	0.059	0.583	0.920
adopted HH occupation: labourer^d^	−0.130	0.302	0.667
adopted HH occupation: landlord^d^	−0.503	0.914	0.582
adopted HH occupation: merchant^d^	−0.148	0.315	0.638
adopted HH occupation: unknown^d^	0.613	0.148	<0.001***
site: Chupei^e^	0.090	0.302	0.766
site: Dajea^e^	−0.976	0.539	0.070^#^
site: Ermei^e^	0.173	0.336	0.607
site: Ettseng^e^	−0.671	0.389	0.085^#^
site: Jibei^e^	−2.268	0.673	0.001***
site: Jiurua^e^	−2.324	0.783	0.003**
site: Lukang^e^	0.337	0.387	0.385
site: Ponhu^e^	0.134	0.341	0.695
site: Taipei^e^	0.447	0.367	0.222
site: Taneia^e^	−1.038	0.353	0.003**
site: Tonka^e^	0.371	0.883	0.675
site: Wujye^e^	0.363	0.325	0.264

^#^*p* ≤ 0.1, *p ≤ 0.05, **p ≤ 0.01, ***p ≤ 0.001.

^a^This analysis conducted on all 3362 women who were adopted to a single household before age 10 and whose marriage outcome is known. No restrictions were applied to their birth year or whether their birth was recorded in the database. The outcome variable is binary, coded 1 if the women married their adoptive brother, 0 otherwise.

^b^Dummy variable coded 1 if the adoption was ever cancelled.

^c^Dummy variable coded 1 if the adoptive family had a biological son at the time of adoption.

^d^Reference category for head household's occupation is agriculture.

^e^Reference category site is Beipu.

### 2.3. Mortality analysis—bivariate models of association

We began by calculating two bivariate measures of mortality. Age-specific mortality rates (ASMRs) were calculated for age groups until the age of 40, after which the number of recorded deaths per category was too low to make any meaningful inferences. ASMRs were calculated as the number of death events recorded for all individuals between age *x* and *x* + 1, divided by the number of person-years of exposure to risk for that interval [39]. ASMRs were calculated separately for biological daughters, ADIL and AD; however, because the time lived by adopted daughters *prior to adoption* was, by definition, not a period of risk for adopted daughters, any person-years lived by a girl before she was adopted were allocated to the person-years for biological daughters.

### 2.4. Inferential analyses of mortality

Inferential models focus on the effects of ADIL adoption versus other forms of adoption on mortality. To account for potential confounding and precision variables (i.e. variables associated with the outcome variable, but not predictors of interest), we first ran a Cox proportional hazard (CPH) model estimating the effect on mortality of having been adopted as ADIL versus as AD. AIC comparisons were used to select covariates and interaction terms included in the model [40]. As, by definition, all girls who experienced adoption had to survive until they were adopted, adoption was treated as the start of the trial period, with age at adoption included as a control (table 5). A time-dependent version of the same model (electronic supplementary material, S1, Table S3) was constructed to examine the effect of parity and time-dependent covariates including age (not age at adoption) and cancellation.

In order to further confirm the causal effect of adoption on mortality, we ran additional Cox models that include adoption and cancellation (*N* = 192; see §2.3 Variables, below) as time-dependent covariates [41]. Whether or not a person was adopted or had their adoption cancelled was coded 0 or 1 for each 1-year time period in their life, allowing us to model the effect of becoming adopted (or having that adoption cancelled) as opposed to simply comparing individuals who were adopted at some point during their lives to those who were not. This circumvents the selection issues that would otherwise exist when comparing adopted with non-adopted individuals (i.e. that arise because death would often precede adoption and be allocated mistakenly to biological daughters).^1^ We ran four models: the first (Model 1, table 2) replicates Mattison *et al*.'s [20] result showing that adopted girls, taken together, were less or equally as likely to die as their biological counterparts, with an additional control for a woman's parity (see below); the second two focus on adopted girls, comparing AD (Model 2, table 3) and ADIL (Model 3, table 4) separately to biological daughters. The final model (4, table 5) compares AD directly to ADIL, *without parity* (see electronic supplementary material, table S3 for results with parity included), as the majority of mortality arose prior to childbearing ages.

**Table 2.** Model 1: Cox proportional hazard model of the hazard of mortality for biological daughters versus all adopted women.^f^

	estimate	exp (Estimate)	s.e.	Pr (>|z|)
age	−0.535	0.586	0.008	<0.001***
age^2^	0.014	1.014	<0.001	<0.001***
adopted^g^	−0.353	0.703	0.066	<0.001***
age × adopted^g^	0.010	1.010	0.006	0.105
cancellation^h^	0.770	2.159	0.370	0.037*
age × cancellation^h^	−0.025	0.975	0.020	0.204
living birth order	0.013	1.013	0.002	<0.001***
parity	−0.202	0.817	0.031	<0.001***
bound feet	−0.042	0.959	0.020	0.033*
head of HH occupation: craftsman^i^	0.009	1.009	0.073	0.902
head of HH occupation: labourer^i^	0.118	1.126	0.033	<0.001***
head of HH occupation: landlord^i^	−0.114	0.892	0.123	0.355
head of HH occupation: merchant^i^	−0.005	0.995	0.039	0.889
head of HH occupation: unknown^i^	0.089	1.093	0.039	0.025*
site: Chupei^e^	0.239	1.269	0.090	0.008**
site: Dajea^e^	0.254	1.289	0.109	0.020*
site: Ermei^e^	−0.160	0.852	0.096	0.095^#^
site: Ettseng^e^	0.334	1.396	0.090	<0.001***
site: Jibei^e^	0.606	1.833	0.099	<0.001***
site: Jiurua^e^	0.090	1.095	0.102	0.375
site: Lukang^e^	0.294	1.342	0.094	0.002**
site: Ponhu^e^	0.374	1.453	0.096	<0.001***
site: Taipei^e^	0.181	1.198	0.094	0.055^#^
site: Taneia^e^	0.265	1.303	0.088	0.003**
site: Tonka^e^	0.151	1.163	0.100	0.132
site: Wujye^e^	0.358	1.430	0.090	<0.001***
birth cohort: 1915^j^	−0.279	0.756	0.073	<0.001***
birth cohort: 1925^j^	−0.527	0.590	0.111	<0.001***
birth cohort: 1935^j^	−0.516	0.597	0.144	<0.001***

^#^*p* ≤ 0.1, *p ≤ 0.05, **p ≤ 0.01, ***p ≤ 0.001.

^a–e^Defined as in table 1.

^f^Sample consists of 31 066 women with 308 645 person-years lived under observation and 9034 death events. All 29 169 biological daughters were included, as well as the 1897 women who were adopted as either AD or ADIL.

^g^Adoption is a time-dependent covariate, coded 1 if the subject was adopted during that year of life, 0 otherwise.

^h^Cancellation is a time-dependent covariate, coded 1 if the subject's adoption was cancelled during that year of life, 0 otherwise.

^i^Time-dependent covariate indicating the occupation of the *current* head of household, i.e. of birth family prior to adoption or subsequent to cancellation, and of adoptive family during adoption.

^j^10-year birth cohorts, reference category 1905–1914.

**Table 3.** Model 2: Cox proportional hazard model of the hazard of mortality for biological daughters versus AD.^k^

	estimate	exp (Estimate)	s.e.	Pr (>|z|)
age	−0.543	0.581	0.008	<0.001***
age^2^	0.015	1.015	<0.001	<0.001***
AD^l^	−0.512	0.599	0.096	<0.001***
age × AD^l^	0.012	1.013	0.010	0.197
cancellation^h^	0.467	1.595	0.590	0.429
age × cancellation^h^	−0.031	0.969	0.034	0.364
living birth order	0.013	1.013	0.002	<0.001***
parity	−0.216	0.806	0.032	<0.001***
bound feet	−0.034	0.966	0.020	0.085^#^
head of HH occupation: craftsman^i^	0.001	1.001	0.073	0.992
head of HH occupation: labourer^i^	0.111	1.117	0.033	0.001**
head of HH occupation: landlord^i^	−0.107	0.898	0.123	0.384
head of HH occupation: merchant^i^	−0.012	0.988	0.039	0.753
head of HH occupation: unknown^i^	0.096	1.100	0.041	0.019*
site: Chupei^e^	0.257	1.294	0.091	0.005**
site: Dajea^e^	0.237	1.267	0.110	0.031*
site: Ermei^e^	−0.142	0.868	0.098	0.146
site: Ettseng^e^	0.323	1.382	0.091	<0.001***
site: Jibei^e^	0.581	1.787	0.100	<0.001***
site: Jiurua^e^	0.075	1.078	0.103	0.465
site: Lukang^e^	0.295	1.343	0.095	0.002**
site: Ponhu^e^	0.357	1.429	0.097	<0.001***
site: Taipei^e^	0.172	1.188	0.095	0.071^#^
site: Taneia^e^	0.251	1.285	0.089	0.005**
site: Tonka^e^	0.144	1.154	0.101	0.155
site: Wujye^e^	0.398	1.489	0.091	<0.001***
birth cohort: 1915^j^	−0.267	0.766	0.075	<0.001***
birth cohort: 1925^j^	−0.507	0.602	0.114	<0.001***
birth cohort: 1935^j^	−0.489	0.613	0.147	0.001**

^#^*p* ≤ 0.1, *p ≤ 0.05, **p ≤ 0.01, ***p ≤ 0.001.

^a–j^Defined as in previous table.

^k^Sample consists of 30 054 women with 291 219 person-years lived under observation and 8809 death events. All 29 169 biological daughters were included, as well as the 885 women who were adopted as AD.

^l^AD is a time-dependent covariate, coded 1 if the subject was adopted as AD during that year of life, 0 otherwise.

**Table 4.** Model 3: Cox proportional hazard model of the hazard of mortality for biological daughters versus ADIL.^m^

	estimate	exp (Estimate)	s.e.	Pr (>|z|)
age	−0.543	0.581	0.008	<0.001***
age^2^	0.014	1.015	<0.001	<0.001***
ADIL^n^	−0.261	0.770	0.083	0.002**
age × ADIL^n^	0.008	1.008	0.007	0.295
cancellation^h^	1.093	2.983	0.473	0.021*
age × cancellation^h^	−0.033	0.968	0.024	0.172
living birth order	0.013	1.013	0.002	<0.001***
parity	−0.201	0.818	0.032	<0.001***
bound feet	−0.039	0.962	0.020	0.048*
head of HH occupation: craftsman^i^	−0.004	0.996	0.073	0.960
head of HH occupation: labourer^i^	0.107	1.113	0.033	0.001**
head of HH occupation: landlord^i^	−0.124	0.883	0.124	0.316
head of HH occupation: merchant^i^	−0.015	0.985	0.039	0.693
head of HH occupation: unknown^i^	0.080	1.083	0.040	0.047*
site: Chupei^e^	0.261	1.298	0.091	0.004**
site: Dajea^e^	0.254	1.289	0.110	0.020*
site: Ermei^e^	−0.146	0.864	0.097	0.133
site: Ettseng^e^	0.326	1.385	0.091	<0.001***
site: Jibei^e^	0.593	1.809	0.100	<0.001***
site: Jiurua^e^	0.083	1.086	0.103	0.420
site: Lukang^e^	0.305	1.357	0.095	0.001**
site: Ponhu^e^	0.353	1.424	0.097	<0.001***
site: Taipei^e^	0.183	1.201	0.095	0.054^#^
site: Taneia^e^	0.259	1.296	0.088	0.003**
site: Tonka^e^	0.148	1.160	0.101	0.141
site: Wujye^e^	0.396	1.485	0.091	<0.001***
birth cohort: 1915^j^	−0.271	0.762	0.075	<0.001***
birth cohort: 1925^j^	−0.533	0.587	0.113	<0.001***
birth cohort: 1935^j^	−0.539	0.583	0.146	<0.001***

^#^*p* ≤ 0.1, *p ≤ 0.05, **p ≤ 0.01, ***p ≤ 0.001.

^a–l^Defined as in previous table.

^m^Sample consists of 30 181 women with 293 900 person-years lived under observation and 8872 death events. All 29 169 biological daughters were included, as well as the 1012 women who were adopted as ADIL.

^n^ADIL is a time-dependent covariate, coded 1 if the subject was adopted as ADIL during that year of life, 0 otherwise.

**Table 5.** Model 4: Cox proportional hazard model of the hazard of mortality for women adopted as AD versus ADIL.^p^

	estimate	exp (Estimate)	s.e.	Pr (>|z|)
ADIL^n^	0.278	1.320	0.141	0.049***
age of adoption	−0.368	0.692	0.071	<0.001***
age of adoption × ADIL^n^	0.080	1.083	0.081	0.324
cancellation^b^	−1.293	0.274	0.351	<0.001***
age of adoption × cancellation^b^	−0.036	0.965	0.181	0.843
bound feet	0.053	1.054	0.175	0.763
living birth order	0.003	1.003	0.013	0.824
head of HH occupation: craftsman^i^	−0.514	0.598	0.721	0.476
head of HH occupation: labourer^i^	0.696	2.006	0.220	0.002***
head of HH occupation: landlord^i^	−0.027	0.973	1.012	0.978
head of HH occupation: merchant^i^	−0.238	0.788	0.402	0.553
head of HH occupation: unknown^i^	0.153	1.166	0.141	0.275
site: Chupei^e^	0.438	1.550	0.461	0.342
site: Dajea^e^	1.359	3.894	1.105	0.219
site: Ermei^e^	−0.249	0.779	0.479	0.602
site: Ettseng^e^	1.018	2.768	0.466	0.029*
site: Jibei^e^	2.248	9.473	0.742	0.002***
site: Jiurua^e^	−0.384	0.681	1.099	0.727
site: Lukang^e^	0.061	1.063	0.523	0.907
site: Ponhu^e^	0.791	2.205	0.468	0.091^#^
site: Taipei^e^	−0.102	0.903	0.637	0.873^#^
site: Taneia^e^	0.745	2.106	0.474	0.116
site: Tonka^e^	0.412	1.510	0.842	0.625
site: Wujye^e^	0.358	1.431	0.473	0.449
birth cohort: 1915^j^	−0.122	0.886	0.129	0.346
birth cohort: 1925^j^	−0.639	0.528	0.153	<0.001***
birth cohort: 1935^j^	−0.520	0.594	0.227	0.022

^#^*p* ≤ 0.1, *p ≤ 0.05, **p ≤ 0.01, ***p ≤ 0.001.

^a–n^Defined as in previous table.

^p^Sample consists of 1897 women and 387 death events, including 885 women adopted as AD and 1012 women adopted as ADIL.

Finally, we include four logistic regression models in the electronic supplementary material (tables S5–S8) that explore the likelihood of ADIL versus AD surviving to age 5 and 10, respectively; these are run on the entire sample of adopted daughters or with girls dying before six months of age excluded as indicated by the table captions. These analyses serve to verify the main results of the CPH models during the period of highest mortality and before childbearing would have begun.

### 2.5. Variables

We include several covariates in our inferential models that are known or hypothesized to be associated with either adoption status or the risk of mortality. The Japanese collected information on the occupation of the head of household, which we use as an indicator of socioeconomic status, lacking any other direct indicators such as household income, land ownership or taxes paid. Following [20], we collapsed over 200 original socioeconomic categories into five: agriculture, craftsman, labourer, landlord and merchant. Labourer and landlord are the most reliable of these categories for indicating wealth, for household heads classified by colonial officers as labourers had no land and no skills and those classified as landlords rented out their land. We also include whether a girl had bound feet, which causes limited mobility, and has been widely (if perhaps mistakenly) believed to be linked to marital aspirations (e.g. [42,43])—for evidence that foot-binding is not linked to marriage, see [44–46]—as well as other indicators of socioeconomic status (e.g. [45–49]). The 10-year birth cohort in which a girl was born is included in our models, as both the prevalence of adoption and the risk of mortality declined through time [14,15,37,50]. Because ADIL prevalence varied regionally in Taiwan (e.g. [37,50]), we include an indicator of the region in which the girl resided. We include parity to account for a possible link between fertility and mortality (including maternal mortality) [51], and to allow for different fertility trajectories for ADIL versus AD [15,52,53]. Finally, we include whether an adoption was cancelled in order to evaluate the effects of cancellation on mortality. Ethnographic insights regarding motivations for cancellation are scant but suggest that cancellations may have occurred when girls ran away or were not delivered to adoptive parents as promised, if a boy (either a biological or adoptive brother) became ill or died (thereby affecting the girl's sibling-based status within the household), or if the girl was sickly. Thus, there are *a priori* reasons to anticipate a positive relationship between cancellations and mortality; hence our inclusion of cancellation as a covariate.

## 3. Results

### 3.1. Identifying DIL adoptions

Using adopted women with known marital outcomes, we found that the presence of an older boy in the adoptive household was the single best predictor of later being married to a boy in the adoptive household (table 1). An adoption being cancelled was the single best negative predictor for being married to a brother in the adoptive household. Consistent with expectations, birth year was also negatively associated with the odds of being married to an adoptive brother, as were the age of adoption (a younger age of adoption was more likely to be associated with ADIL status), having bound feet, and residing in areas where the prevalence of minor marriage was known to be low (see [20], electronic supplementary material, table S1).

Applying this predictive method to our sample of adopted women with known marital outcomes, we found that *not* having a boy present in the adoptive household at the time of adoption correctly predicted marrying outside of the adoptive family 74.2% of the time, while having at least one such adoptive brother at the time of adoption correctly predicted that an individual would marry one of her adoptive brothers 94.8% of the time. On eliminating girls whose adoption was subsequently cancelled, the predictive accuracy was 80.0% and 94.9%, respectively. These values were consistent enough to confirm Arthur Wolf's rule (see §2.2, above); thus, we identified as ADIL any adopted daughters who had an adoptive brother at the time of adoption and whose adoption was not cancelled.

### 3.2. Analyses of mortality

**Figure 1.**
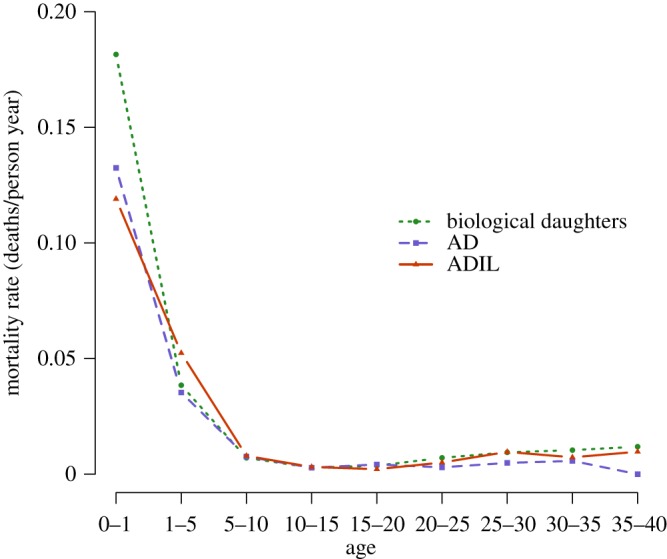
Age-specific mortality rates of biological daughters, AD and ADIL. To be classified as AD or ADIL, girls must survive long enough to be adopted, probably accounting at least partially for low mortality rates of adopted daughters in the first year of life; person-years have been allocated according to the timing of adoption; see main text for details.

Age-specific mortality rates (figure 1; electronic supplementary material, tables S1 and S2) show that, contrary to expectations, ADIL had *higher* mortality than AD, although both categories of adopted daughters appeared to fare better than biological daughters. ADIL showed higher mortality particularly between the ages of 1 and 5 (figure 1), the period where most of the adoptions were occurring. Although mortality rates for AD are slightly higher than for ADIL during the first year of life, this is based on a small sample because few person-years were lived by adopted daughters in that age category. From age 5 to age 15, rates are relatively similar; mortality appears to rise again for biological daughters and ADIL relative to AD during the childbearing years; these results must be interpreted with caution given small sample sizes at later ages. We include parity as a control in our subsequent analyses except as otherwise noted to allow for the possibility that differential fertility drives differences in mortality during adulthood.

Cox proportional hazard (CPH) models using time-dependent covariates confirm that ADIL fared better than biological daughters with respect to mortality, but worse than AD. In particular, Model 1 (table 2) confirms the protective effect of adoption described by Mattison *et al*. [20], with adopted girls having a 29.7% lower hazard of mortality at any given time than biological daughters raised in their natal households. Models 2 and 3 (tables 3 and 4) replicate these results with AD and ADIL, respectively, and the protective effect of adoption is much stronger for AD (*Β *= −0.51) than for ADIL (*Β *= −0.26). Comparing AD directly with ADIL reveals a similar trend: AD have lower mortality than ADIL (table 5), though, due to small sample size, this result is attenuated with the addition of parity as a covariate (electronic supplementary material, table S3).

Importantly, Model 3 (table 4) also reveals that, for ADIL, having an adoption cancelled is extremely detrimental with respect to mortality: an ADIL whose adoption was cancelled had a 1.98 higher hazard of dying than a girl who never experienced adoption. This increased probability of mortality suggests strongly that adoption, *per se*, is protective against mortality. Sickliness of girls whose adoption was cancelled seems an unlikely explanation of their higher mortality given that only six cases were observed to die soon after (within 30 days of) cancellation.

**Figure 2.**
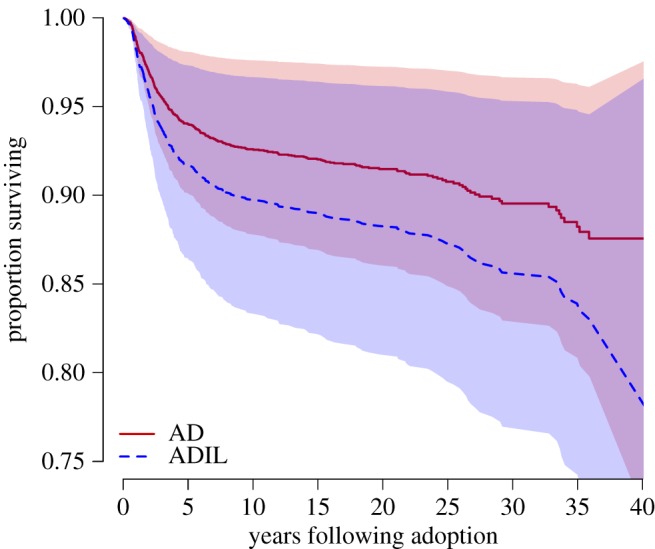
Predicted survivorship for AD (red; solid) and ADIL (blue; dashed), controlling for covariates in Model 4 (table 5). Highlighted regions indicate 95% confidence intervals.

All of the analyses except one point to AD having higher survivorship than ADIL. In a direct comparison that excludes parity as a covariate (Model 4, table 5), AD have approximately 32% lower hazard of mortality than ADIL, controlling for other covariates. Figure 2 displays these differences graphically in terms of survivorship. By 40 years after adoption, 83.6% of the adopted daughters were still alive, whereas only 77.9% of adopted daughters-in-law were. Adding parity as a time-dependent covariate to the comparison of AD and ADIL results in non-significance. We strongly suspect that this is an issue of sample size. Four logistic regression models comparing the probability of surviving to age 5 or age 10 (see electronic supplementary material, tables S5–S8) support lower survivorship of ADIL compared to AD. The effect is the strongest to age 5, in line with mortality differences being largest at early ages (figure 1 and electronic supplementary material, tables S5–S8).

Finally, in all models, covariates' associations with mortality are fundamentally in line with expectations. Girls born at later parities suffered higher mortality, as did daughters of labourers. The north–south gradient in mortality is also apparent in these data. Interestingly, having bound feet is also protective against mortality, though the magnitude of this effect is much smaller than the effect of adoption in all models. Finally, a woman's parity shows an inverse association with the hazard of mortality in all models; i.e. women with higher realized fertility survived longer than those with lower fertility. This effect was apparent for all classes of daughter.

## 4. Discussion

In this study, we present a finely resolved test of the effects of adoption on mortality, using a systematic rule to identify girls who were likely to have been adopted for the purposes of minor marriage versus for other purposes. Although inspired by ethnographic evidence, our rule was verified by demonstrated associations between a girl's natal and adoptive circumstances and her subsequent marital outcome. Thus, whereas we showed previously that adopted girls on the whole fared better than never-adopted girls in terms of survivorship, in this paper, we delineate more precisely the effects of different *forms* of adoption on mortality. Specifically, both ADIL and AD experienced lower mortality than biological daughters who remained in the care of their natal families, but this effect was stronger among AD than among ADIL. While ADIL did indeed experience lower mortality than biological daughters (Model 3, table 4), the association between adoption and mortality was more strongly negative for AD (Model 2, table 3). This trend is upheld in a direct comparison of AD to ADIL (Model 4, table 5), despite small sample size, though this trend loses significance with the addition of parity as a time-dependent covariate (electronic supplementary material, table S3). Taken together, these results reinforce (i) the importance of the motivations for different types of adoption in identifying its likely consequences in terms of life history and (ii) the need to look beyond narrow kin selection (i.e. the focus on the coefficient of relatedness) as the main evolutionary explanation for patterns of human adoption and its life-history consequences; other costs and benefits that might accrue to adoptive families should also be considered [5,6,13,54].

Whereas the majority of research in this area has focused on broad comparisons between biological and non-biological children (e.g. [20–22]), we have shown that the specific *type* of adoption affected the risk of mortality for Taiwanese girls during the early twentieth century. In reviewing the effects of fosterage on child welfare, Castle [55] points to the importance of adoptive parental volition in mediating foster children's nutritional outcomes—children who were demanded by their foster parents had better nutritional outcomes than children who were not. Similarly, Pillai & Sharma [26] contend that fosterage, *per se*, is less important than the contexts surrounding fosterage in affecting child outcomes: crisis situations lead to worse outcomes than voluntary fosterage. In the historical Taiwanese case, AD were explicitly sought by their adoptive parents to remedy perceived demographic deficiencies; thus, we might anticipate that their welfare should not have suffered as much as children being adopted under more dubious circumstances. Indeed, AD were adopted into households with an average of 0.25 adoptive siblings, compared to 3.36 for ADIL, consistent with AD serving as stand-ins when biological children were lacking.

That AD out-survive ADIL is not in line with kin selection explanations [20] that anticipate preferential treatment of the latter given their direct contributions to the adoptive parents' inclusive fitness, leaving us to speculate as to what drove the differences in mortality shown here. The prevalence of in-family adoption in this dataset is exceedingly small (electronic supplementary material, figure S1) and such adoptions were not considered in our analyses. Thus, the protective effect of adoption is not due to increased altruism towards genetically related individuals. One possibility is that adopted daughters, regardless of category of adoption, provided economic or other benefits to their adoptive families that were not readily available via other members of the household [14,56]. Given the early position in the sibset into which these girls were adopted, adopted daughters could have assisted with activities (e.g. childcare) that increased the fertility of their adoptive parents [14,15,38,39,54,57,58]. A second possibility is that adopting a daughter at a relatively young age—particularly if daughters were breastfed, as often happened in the case of ADIL—invoked a psychological response in their adoptive parents [59] that improved the treatment of adopted daughters relative to daughters who remained in their natal homes. This hypothesis seems unlikely given that cancelled adoptions were associated with higher mortality and that Wolf [38] has provided evidence from the colonial Taiwan household registers against breastfeeding as mediating the relationship between adoption and mortality. Moreover, a presumed breastfeeding mediation of mortality would not explain why biological daughters fared worse than adopted daughters or why AD fared better than ADIL, given similar distributions in the age of adoption (electronic supplementary material, figure S2).

A third possibility worth serious consideration is that parents neglected their biological daughters. Ethnographic materials suggest that biological daughters who remained at home were viewed negatively. Seen as expensive (because they required dowries) and as a means by which other people's lineages were perpetuated, unless they began making economic contributions at an early age [45,46,48], biological daughters might have seemed like poor investments relative to giving one's own biological daughters up for adoption to another family. Although many have emphasized the stigma and neglect or mistreatment of adopted children in Taiwan during this period, treatment of biological children up until recently was also often harsh [60]. Disobedience of children was often met by beatings, because ‘rods produce filial [children]’ [58, p. 158]. Moreover, although adopted children were also routinely beaten by their Taiwanese parents, the number of deaths arising due to such beatings was probably very small [58]. Comparing our age-specific mortality rates to mortality rates drawn from the UN model life tables (Far Eastern female pattern; highest mortality; electronic supplementary material, figure S3) shows that AD and ADIL track the modelled mortality better than biological daughters, whose mortality appears slightly inflated, providing somewhat indirect support for the neglect of biological daughters. This result is consistent with models of conflict among kin wherein biological kin may be disfavoured if they compete for scarce resources [61]. Moreover, the voluntary nature of adoption in this context might be expected to lead to better outcomes for adopted children [62].

A final possibility arises due to potential differences in fertility between different classes of adopted daughter and biological daughters. In line with Westermarck's hypothesis that individuals co-reared together from young ages experience incest avoidance [14,15,38,52–54,63], ADIL have been shown to have lower fertility than biological daughters who marry individuals from outside their household [14,15,38,52–54,63,64]. If higher fertility results in higher mortality [65], including maternal mortality, then biological daughters' excess mortality might be due simply to their higher parity. Similarly, ADIL might suffer higher mortality than AD if AD had lower fertility. Our models suggest that this possibility is not a likely explanation for these data. The differences in mortality that we report exist with and without inclusion of a woman's parity as a control variable. In fact, parity shows an inverse association with mortality, suggesting that women with higher fertility also survive longer. Phenotypic correlation (i.e. women with higher energetic reserves reproduce more *and* have higher survivorship) has been proposed to account for positive correlations between fertility and survivorship [66,67]. A strong test of how fertility relates to mortality would have to consider not only the pace of reproduction and maternal energy budgets [66], but also potential cultural effects, such as Wolf's findings that colonial Taiwanese women's risk of divorce—with loss of old-age support implied—only declined significantly after giving birth to multiple sons [14,15]. Such a test is beyond the scope of this paper and, given that the strongest divergences in mortality arise early in life [8], is unlikely to shed additional light on why AD and ADIL experience lower mortality than biological daughters.

That adopted daughters (ADIL and AD) experienced lower mortality if their feet were bound is an interesting result that we did not anticipate. Prior work on the functions of bound feet has focused on their use as a marker of status [42,43], as a means of insuring paternity (e.g. [68]), or as a way to increase the contributions of women to household economics through handicraft production [44–49]. Any of these functions could also be associated with decreased mortality if adoptive parents felt that the benefits of adoption were increased by the binding of their adopted daughters' feet. In this dataset, bound feet may also serve as an ethnic marker, because Sinicized plains Aborigines were both unlikely to have bound feet and to practise minor marriage [12]. Regardless, our result implies that functional approaches to understanding the benefits and costs of bound feet may, therefore, be more illuminating than those focused solely on its (highly visible) costs.

Our data and analyses are subject to several important limitations. First, our measures of socioeconomic status are imperfect and do not allow for fine-grained resolution of the confounding effects of socioeconomic status on adoption and mortality. Although such effects may have been relatively weak given the ubiquity of adoption across socioeconomic differentiation [14,15], we may have underestimated the importance of socioeconomic status in mediating the relationship between adoption and mortality. However, we find no evidence that different types of adoption occurred at different rates to parents of different socioeconomic status (electronic supplementary material, table S4) and higher rates of mortality among children of labourers is consistent with their disadvantaged status in Taiwan during this time period [12,14,37]. Second, we lost a significant portion of the sample by excluding individuals whose births were not registered in the dataset and by including only cases of adoption where circumstances at birth *and* adoption were known (as described in electronic supplementary material). These restrictions may limit generalizability of our findings if the characteristics of girls who could be followed to adoptive households differed from those who could not be followed. Still, few datasets are able to address the contributions of adoption to health outcomes in longitudinal fashion, let alone while taking characteristics of the natal household into account.

## 5. Conclusion

We have shown that adopted daughters—both ADIL and AD—experienced lower mortality than biological daughters remaining in their natal household in early twentieth-century Taiwan, but that AD—who did not contribute to the perpetuation of the adoptive parents' lineage—out-survived ADIL. These findings suggest that the functions of adoption and other forms of allocare should be viewed in the context of forms of cooperation that extend beyond the narrowest version of the kin selection hypothesis. In revealing lower mortality for AD than for ADIL, this study contradicts the hypothesis that ADIL should be favoured because they contribute to biological perpetuation of the lineage, suggesting that other unexplored benefits, such as enhancement of parental fertility, may accrue to adoptive parents or alternatively that biological daughters may be neglected, perhaps because they compete for scarce resources, which are delivered instead to biological sons.

Our findings point to the importance of the specific contexts that give rise to adoption—especially the differences between adoption in industrialized and non-industrialized populations—if we are to make any general statements about its functional significance. Adoption practices vary significantly within and among populations, from Western-style adoption of non-related children [69], often to nulliparous parents, to adoption of closely related kin as a means of redistributing children to optimize household size and labour inputs [10,70]. Our study thus urges renewed attention in at least three domains of evolutionary research on adoption. First, our study shows that ‘adoption’ is not monolithic and that the specific forms it takes can differ and have different consequences in terms of individual life histories. Second, the cultural contexts under which adoption occurs will affect the attendant costs and benefits and must be considered in evolutionary arguments describing the adaptive value of adoption in any given time or place [3,7]. Third, our study offers the interesting possibility that biological daughters may be disfavoured relative to adopted children who are intentionally brought in to serve various roles in the adoptive family. Conflict among kin is widely recognized to create biases in parental investment [61]; extensions of such models to explaining the differences in care of biological and non-biological children [71,72] would facilitate a more nuanced understanding of whether and how parents ultimately stand to benefit from the investments they make in their children, related or otherwise.

## Ethics

Data are obtained from the Academia Sinica and used in accordance with their policies.

## Supplementary Material

Electronic Supplementary Material - Sample selection and additional analyses

## Footnotes

1 NB: This selection bias does not affect the comparison of different forms of adoption to each other as these girls are followed from the point of adoption forward.

## Data accessibility

The dataset underlying this study includes the full household registers from 13 sites distributed across Taiwan (all are listed in the manuscript tables) and includes two files per site: a ‘static’ file that includes information on dates of birth, dates of death, ID numbers of the mother and father, the sex, the same-sex sibling order, and whether the individual had bound feet; the second file for each site is a ‘dynamic’ file that includes information on events (we were concerned with adoption, cancellation of adoption and marriage) that occurred during the individual's lifetime and the dates of these events as well as relevant IDs (partner ID, adoptive parent ID).

Providing a full dataset would both run the risk of identifying individuals involved in the study and would be in violation of the terms of our access to the dataset provided by Academia Sinica. Academia Sinica is an institution of higher learning in Taiwan (which is analogous to NIH+NAS); their programme for historical demography maintains the registers and provides access to researchers based on the specific needs of each researcher. Access is granted through Academia Sinica's application form, which is available here (http://www.demography.sinica.edu.tw/Downloads/2202/db/THHRD%20Application%20and%20Regulation%20-EN.pdf). There is no independent deposition of raw data allowed under their policy.

To assist the editor and referees in assessing this work and associated data, we provided temporary access to the dataset that we worked with to run the analyses to editors and reviewers as a password-protected file that accompanied this submission. Furthermore, we provide a range of data in aggregate (e.g. means, variances and ranges) that might be of interest here: http://bit.ly/2lTXFuN. Additional data may be requested on an individual basis via the above Academia Sinica link.
