# Supplementary material for: Adopted daughters and adopted daughters-in-law in Taiwan: a mortality analysis
Source: R Soc Open Sci. 2018 Mar 21;5(3):171745. doi: 10.1098/rsos.171745 (PMC5882702; doi:10.1098/rsos.171745)
Supplement: Electronic Supplementary Material - Sample selection and additional analyses [file rsos171745supp1.docx]

**Electronic Supplementary Material**

Additional Information on Sample Selection (Main Text 2.1)

The original sample included 75,675 women, including 21,511 who had been adopted, and of whom 19,409 died. We excluded from these women 1,406 who were adopted after age ten, 507 who were adopted multiple times, 53 who were adopted into the same household that they left, 5,337 who were adopted out but whose adoptive parents were unknown, and 3,336 for whom we had no information on the adoptive mother. The remainder were lost because they had been adopted by a closer relative, were born outside the study area, or were born prior to 1905, leaving us with a final sample of 31,066 girls, of whom 1,897 were adopted.

|  |
| --- |
| **Figure S1. Adoptions among close kin.** The overall fraction of close-kin adoptions was small (423 children; 1.51% of 25,122 adopted and 0.30% of 140,324 total). Of these, boys were most frequently adopted by their paternal uncles, possibly reflecting similar trends elsewhere in China during this time period (Watson, 1975). |

| 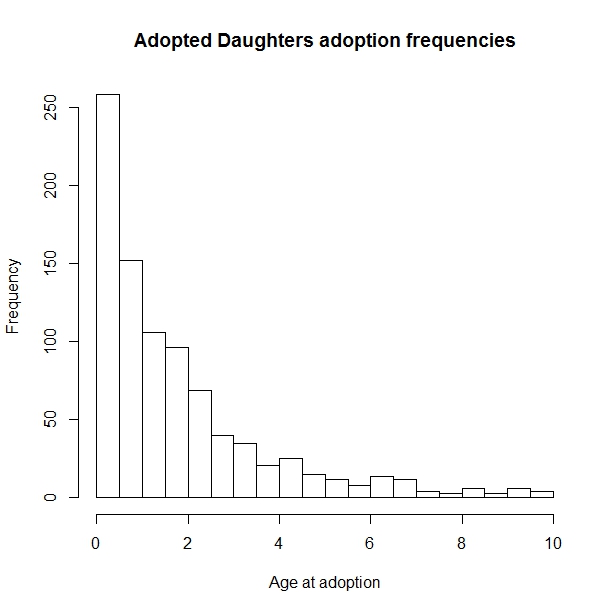 | 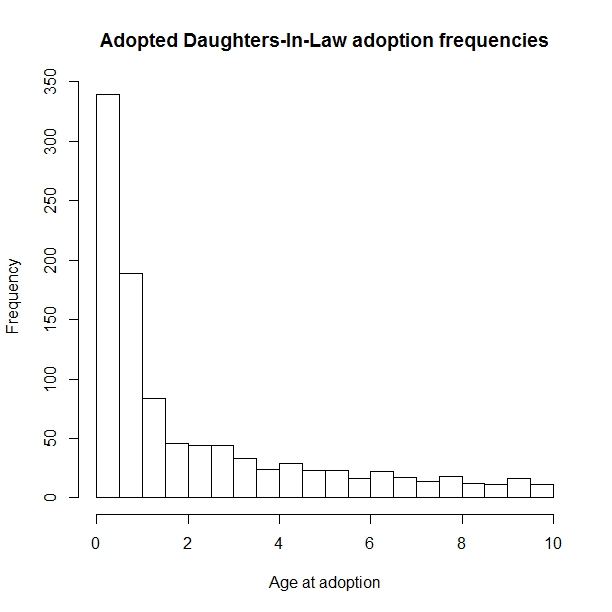 |
| --- | --- |
| **Figure S2. Frequency distributions for age of adoption for AD (left) and ADIL (right).** Note that the y-axis differs. | |

|  |
| --- |
| **Figure S3. Corrected ASMRs by class of daughter alongside model UN mortality rate.** Pattern is Far Eastern, highest mortality, female. |

**Table S1.** Age-specific mortality rates^a^ by adoptive status of daughter in 1-year cohorts

| Age Categories | Biological Daughters | Adopted Daughters | Adopted Daughters-In-Law |
| --- | --- | --- | --- |
| 0-1 | 0.182 | 0.132 | 0.119 |
| 1-2 | 0.066 | 0.064 | 0.100 |
| 2-3 | 0.038 | 0.044 | 0.062 |
| 3-4 | 0.025 | 0.033 | 0.031 |
| 4-5 | 0.017 | 0.009 | 0.025 |
| 5-6 | 0.010 | 0.015 | 0.013 |
| 6-7 | 0.008 | 0.008 | 0.011 |
| 7-8 | 0.007 | 0.009 | 0.007 |
| 8-9 | 0.004 | 0.005 | 0.004 |
| 9-10 | 0.004 | 0.002 | 0.004 |
| 10-11 | 0.002 | 0 | 0.004 |
| 11-12 | 0.003 | 0.003 | 0.006 |
| 12-13 | 0.003 | 0.005 | 0 |
| 13-14 | 0.003 | 0 | 0.003 |
| 14-15 | 0.003 | 0.006 | 0.002 |
| 15-16 | 0.003 | 0.010 | 0 |
| 16-17 | 0.003 | 0.002 | 0.002 |
| 17-18 | 0.004 | 0 | 0.004 |
| 18-19 | 0.004 | 0.006 | 0.002 |
| 19-20 | 0.005 | 0 | 0.003 |
| 20-21 | 0.005 | 0 | 0 |
| 21-22 | 0.008 | 0.004 | 0.010 |
| 22-23 | 0.009 | 0 | 0.004 |
| 23-24 | 0.007 | 0.005 | 0.004 |
| 24-25 | 0.007 | 0.006 | 0.009 |
| 25-26 | 0.011 | 0 | 0.010 |
| 26-27 | 0.007 | 0.015 | 0.011 |
| 27-28 | 0.012 | 0 | 0.012 |
| 28-29 | 0.005 | 0.009 | 0.007 |
| 29-30 | 0.012 | 0 | 0.007 |
| 30-31 | 0.013 | 0 | 0 |
| 31-32 | 0.014 | 0 | 0 |
| 32-33 | 0.003 | 0 | 0.009 |
| 33-34 | 0.014 | 0.035 | 0.020 |
| 34-35 | 0.006 | 0 | 0.012 |
| 35-36 | 0.011 | 0 | 0.030 |
| 36-37 | 0.007 | 0 | 0 |
| 37-38 | 0.019 | 0 | 0 |
| 38-39 | 0 | 0 | 0 |
| 39-40 | 0.036 | 0 | 0 |
| 40+ | 0 | 0 | 0.225 |

^a^ Person-years have been allocated according to time of adoption such that person-years lived prior to adoption are allocated as periods of risk for biological daughters; see main text for details.

**Table S2.** Age-specific mortality rates^a^ by adoptive status of daughter in 5-year cohorts.

| Age | Biological Daughters | AD | ADIL |
| --- | --- | --- | --- |
| 0-1 | 0.18 | 0.13 | 0.12 |
| 1-5 | 0.04 | 0.04 | 0.05 |
| 5-10 | 0.01 | 0.01 | 0.01 |
| 10-15 | 0.00 | 0.00 | 0.00 |
| 15-20 | 0.00 | 0.00 | 0.00 |
| 20-25 | 0.01 | 0.00 | 0.01 |
| 25-30 | 0.01 | 0.00 | 0.01 |
| 30-35 | 0.01 | 0.01 | 0.01 |
| 35-40 | 0.01 | 0.00 | 0.01 |
| 40-45 | 0.00 | 0.00 | 0.22 |

^a^ Person-years have been allocated according to time of adoption such that person-years lived prior to adoption are allocated as periods of risk for biological daughters; see main text for details.

**Table S3.** Model S1: Cox Proportional Hazard model of the effects of being adopted as ADIL vs AD on hazard of mortality (controlling for parity and time-dependent covariates)^*a^

|  | **Estimate** | **exp(Estimate)** | **Std. Error** | **Pr (>\|z\|)** |  |
| --- | --- | --- | --- | --- | --- |
| **Age** | -0.311 | 0.733 | 0.031 | < 0.001 | *** |
| **Age^2^** | 0.008 | 1.008 | 0.001 | < 0.001 | *** |
| **ADIL^b^** | 0.233 | 1.262 | 0.129 | 0.071 | . |
| **Age x ADIL^b^** | 0.009 | 1.009 | 0.014 | 0.513 |  |
| **Cancellation^c^** | 0.997 | 2.711 | 0.428 | 0.020 | * |
| **Age x Cancellation^c^** | -0.018 | 0.982 | 0.025 | 0.474 |  |
| **Living birth order** | 0.005 | 1.005 | 0.012 | 0.665 |  |
| **Parity** | -0.029 | 0.971 | 0.102 | 0.776 |  |
| **Bound feet** | -0.075 | 0.928 | 0.160 | 0.640 |  |
| **Head of HH occupation: craftsman^d^** | -0.303 | 0.739 | 0.748 | 0.686 |  |
| **Head of HH occupation: laborer^d^** | 0.569 | 1.767 | 0.207 | 0.006 | ** |
| **Head of HH occupation: landlord^d^** | -0.439 | 0.645 | 1.009 | 0.664 |  |
| **Head of HH occupation: merchant^d^** | -0.266 | 0.767 | 0.378 | 0.482 |  |
| **Head of HH occupation: unknown^d^** | 0.867 | 2.379 | 0.139 | < 0.001 | *** |
| **Site: Chupei^e^** | 0.414 | 1.513 | 0.461 | 0.370 |  |
| **Site: Dajea^e^** | 0.842 | 2.321 | 1.139 | 0.460 |  |
| **Site: Ermei^e^** | -0.252 | 0.777 | 0.478 | 0.598 |  |
| **Site: Ettseng^e^** | 1.014 | 2.758 | 0.465 | 0.029 | * |
| **Site: Jibei^e^** | 2.780 | 16.114 | 0.746 | < 0.001 | *** |
| **Site: Jiurua^e^** | -0.499 | 0.607 | 1.099 | 0.650 |  |
| **Site: Lukang^e^** | 0.101 | 1.106 | 0.523 | 0.847 |  |
| **Site: Ponhu^e^** | 0.957 | 2.603 | 0.467 | 0.041 | * |
| **Site: Taipei^e^** | 0.056 | 1.057 | 0.635 | 0.930 |  |
| **Site: Taneia^e^** | 0.761 | 2.141 | 0.473 | 0.107 |  |
| **Site: Tonka^e^** | 0.367 | 1.443 | 0.843 | 0.663 |  |
| **Site: Wujye^e^** | 0.119 | 1.127 | 0.472 | 0.800 |  |
| **Birth cohort: 1915^f^** | -0.138 | 0.871 | 0.247 | 0.575 |  |
| **Birth cohort: 1925^f^** | -0.204 | 0.816 | 0.431 | 0.637 |  |
| **Birth cohort: 1935^f^** | -0.077 | 0.926 | 0.598 | 0.898 |  |

^a^ Sample consists of 1,897 women with a total of 32,171 person-years lived under observation and 387 recorded death events.

^b^ ADIL is a time-dependent covariate, coded 1 if the subject was adopted as ADIL during that year of life, 0 otherwise.

^c^ Cancellation is a time-dependent covariate, coded 1 if the subject’s adoption was cancelled during that year of life, 0 otherwise.

^d^ Reference category for head household’s occupation is agriculture.

^e^ Reference category site is *Beipu*.^f^ 10-year birth cohorts, reference category 1905-1914.

**Table S4.** Distribution of adoption type by socio-economic status (head of household's occupation)^a^.

|  | Birth Father | | Adoptive Father | |
| --- | --- | --- | --- | --- |
|  | AD | ADIL | AD | ADIL |
| **Agriculture** | 61.06 % | 62.71% | 21.14% | 16.05% |
| **Craftsman** | 2.37% | 1.13% | 0.89% | 0.56% |
| **Laborer** | 15.71% | 15.71% | 0.00% | 4.86% |
| **Landlord** | 0.69% | 0.45% | 4.64% | 0.45% |
| **Merchant** | 9.78% | 12.09% | 3.16% | 1.69% |
| **Unknown** | 10.38% | 7.91% | 70.16% | 76.38% |
| **Total** | 100.00% | 100.00% | 100.00% | 100.00% |

^a^ n = 1,897; including 1,012 girls adopted as AD and 885 girls adopted as ADIL.

**Table S5.** Logistic regression model predicting probability of survival to age 5 for ADIL compared to AD. (Includes girls who died before 6 months of age)^ab^

|  | **Estimate** | **Std. Error** | **Pr(>\|z\|)** |  |
| --- | --- | --- | --- | --- |
| **(Intercept)** | 1.124 | 0.566 | 0.047 | * |
| **ADIL** | -0.391 | 0.150 | 0.009 | ** |
| **Age adopted** | 0.764 | 0.088 | < 0.001 | *** |
| **Birth cohort: 1915** | -0.007 | 0.183 | 0.969 |  |
| **Birth cohort: 1925** | 0.631 | 0.203 | 0.002 | ** |
| **Birth cohort: 1935** | 0.343 | 0.276 | 0.215 |  |
| **Site: Chupei** | -0.194 | 0.534 | 0.716 |  |
| **Site: Dajea** | -1.527 | 1.741 | 0.380 |  |
| **Site: Ermei** | 0.694 | 0.574 | 0.227 |  |
| **Site: Ettseng** | -0.759 | 0.546 | 0.165 |  |
| **Site: Jibei** | -1.590 | 1.394 | 0.254 |  |
| **Site: Jiurua** | 12.873 | 344.011 | 0.970 |  |
| **Site: Lukang** | 0.695 | 0.651 | 0.286 |  |
| **Site: Ponhu** | -0.189 | 0.548 | 0.730 |  |
| **Site: Taipei** | 0.464 | 0.759 | 0.541 |  |
| **Site: Taneia** | -0.461 | 0.557 | 0.407 |  |
| **Site: Tonka** | -0.744 | 1.065 | 0.485 |  |
| **Site: Wujye** | -0.284 | 0.553 | 0.607 |  |
| **Birth HH occupation: craftsman** | -0.115 | 0.498 | 0.818 |  |
| **Birth HH occupation: laborer** | -0.449 | 0.206 | 0.029 | * |
| **Birth HH occupation: landlord** | -1.187 | 0.827 | 0.151 |  |
| **Birth HH occupation: merchant** | 0.102 | 0.267 | 0.702 |  |
| **Birth HH occupation: unknown** | 0.121 | 0.357 | 0.735 |  |
| **Adopted HH occupation: craftsman** | 0.907 | 1.121 | 0.419 |  |
| **Adopted HH occupation: laborer** | -0.619 | 0.333 | 0.063 | . |
| **Adopted HH occupation: landlord** | -0.728 | 1.341 | 0.587 |  |
| **Adopted HH occupation: merchant** | 0.485 | 0.601 | 0.419 |  |
| **Adopted HH occupation: unknown** | -0.069 | 0.196 | 0.723 |  |

^a^ Sample consists of 3,362 women, with a recorded birth after 1905, adopted to unrelated families of known identities only once and before the age of 10.

^b^ Variable notes are the same as in Table S3

**Table S6.** Logistic regression model predicting probability of survival to age 10 for ADIL compared to AD. (Includes girls who died before 6 months of age)^ab^

|  | **Estimate** | **Std. Error** | **Pr(>\|z\|)** |  |
| --- | --- | --- | --- | --- |
| **(Intercept)** | 1.280 | 0.555 | 0.021 | * |
| **ADIL** | -0.343 | 0.141 | 0.015 | * |
| **Age adopted** | 0.502 | 0.058 | < 0.001 | *** |
| **Birth cohort: 1915** | 0.064 | 0.171 | 0.710 |  |
| **Birth cohort: 1925** | 0.654 | 0.188 | 0.001 | *** |
| **Birth cohort: 1935** | -0.753 | 0.297 | 0.011 | * |
| **Site: Chupei** | -0.478 | 0.528 | 0.365 |  |
| **Site: Dajea** | -1.339 | 1.514 | 0.376 |  |
| **Site: Ermei** | 0.715 | 0.568 | 0.208 |  |
| **Site: Ettseng** | -1.189 | 0.539 | 0.028 | * |
| **Site: Jibei** | -16.301 | 771.295 | 0.983 |  |
| **Site: Jiurua** | 12.968 | 363.607 | 0.972 |  |
| **Site: Lukang** | 0.513 | 0.638 | 0.421 |  |
| **Site: Ponhu** | -0.610 | 0.542 | 0.261 |  |
| **Site: Taipei** | 0.448 | 0.756 | 0.554 |  |
| **Site: Taneia** | -0.681 | 0.549 | 0.215 |  |
| **Site: Tonka** | -0.678 | 1.024 | 0.508 |  |
| **Site: Wujye** | -0.360 | 0.544 | 0.509 |  |
| **Birth HH occupation: craftsman** | -0.280 | 0.454 | 0.538 |  |
| **Birth HH occupation: laborer** | -0.599 | 0.192 | 0.002 | ** |
| **Birth HH occupation: landlord** | -1.308 | 0.827 | 0.114 |  |
| **Birth HH occupation: merchant** | 0.175 | 0.258 | 0.498 |  |
| **Birth HH occupation: unknown** | -0.215 | 0.350 | 0.539 |  |
| **Adopted HH occupation: craftsman** | 1.190 | 1.116 | 0.286 |  |
| **Adopted HH occupation: laborer** | -0.543 | 0.325 | 0.095 | . |
| **Adopted HH occupation: landlord** | -0.260 | 1.295 | 0.841 |  |
| **Adopted HH occupation: merchant** | 0.583 | 0.589 | 0.322 |  |
| **Adopted HH occupation: unknown** | -0.040 | 0.183 | 0.829 |  |

^a^ Sample consists of 1,687 women, with a recorded birth after 1905, adopted to unrelated families of known identities only once and before the age of 10.

^b^ Variable notes are the same as in Table S3

**Table S7.** Logistic regression model predicting probability of survival to age 5 for ADIL compared to AD. (Does not include girls who died before 6 months of age)^ab^

|  | **Estimate** | **Std. Error** | **Pr(>\|z\|)** |  |
| --- | --- | --- | --- | --- |
| **(Intercept)** | 1.306 | 0.607 | 0.031 | * |
| **ADIL** | -0.394 | 0.152 | 0.010 | ** |
| **Age adopted** | 0.728 | 0.086 | < 2e-16 | *** |
| **Birth cohort: 1915** | 0.027 | 0.186 | 0.883 |  |
| **Birth cohort: 1925** | 0.651 | 0.207 | 0.002 | ** |
| **Birth cohort: 1935** | 0.336 | 0.277 | 0.226 |  |
| **Site: Chupei** | -0.312 | 0.575 | 0.588 |  |
| **Site: Dajea** | 12.033 | 1064.063 | 0.991 |  |
| **Site: Ermei** | 0.626 | 0.617 | 0.310 |  |
| **Site: Ettseng** | -0.899 | 0.587 | 0.125 |  |
| **Site: Jibei** | -1.742 | 1.409 | 0.216 |  |
| **Site: Jiurua** | 13.730 | 571.215 | 0.981 |  |
| **Site: Lukang** | 0.486 | 0.686 | 0.479 |  |
| **Site: Ponhu** | -0.287 | 0.591 | 0.626 |  |
| **Site: Taipei** | 0.237 | 0.789 | 0.764 |  |
| **Site: Taneia** | -0.576 | 0.597 | 0.335 |  |
| **Site: Tonka** | -0.925 | 1.073 | 0.389 |  |
| **Site: Wujye** | -0.433 | 0.593 | 0.465 |  |
| **Birth HH occupation: craftsman** | -0.144 | 0.497 | 0.772 |  |
| **Birth HH occupation: laborer** | -0.381 | 0.211 | 0.071 | . |
| **Birth HH occupation: landlord** | -1.174 | 0.819 | 0.152 |  |
| **Birth HH occupation: merchant** | 0.142 | 0.272 | 0.602 |  |
| **Birth HH occupation: unknown** | 0.058 | 0.360 | 0.871 |  |
| **Adopted HH occupation: craftsman** | 0.844 | 1.119 | 0.451 |  |
| **Adopted HH occupation: laborer** | -0.540 | 0.345 | 0.118 |  |
| **Adopted HH occupation: landlord** | -0.759 | 1.330 | 0.568 |  |
| **Adopted HH occupation: merchant** | 0.379 | 0.599 | 0.527 |  |
| **Adopted HH occupation: unknown** | -0.093 | 0.200 | 0.640 |  |

^a^ Sample consists of 1,817 women, with a recorded birth after 1905, adopted to unrelated families of known identities only once and before the age of 10.

^b^ Variable notes are the same as in Table S3

**Table S8.** Logistic regression model predicting probability of survival to age 10 for ADIL compared to AD. (Does not include girls who died before 6 months of age)^ab^

|  | **Estimate** | **Std. Error** | **Pr(>\|z\|)** |  |
| --- | --- | --- | --- | --- |
| **(Intercept)** | 1.462 | 0.596 | 0.014 | * |
| **ADIL** | -0.338 | 0.143 | 0.018 | * |
| **Age adopted** | 0.480 | 0.057 | < 0.001 | *** |
| **Birth cohort: 1915^b^** | 0.092 | 0.174 | 0.595 |  |
| **Birth cohort: 1925** | 0.668 | 0.191 | 0.000 | *** |
| **Birth cohort: 1935** | -0.760 | 0.298 | 0.011 | * |
| **Site: Chupei** | -0.613 | 0.570 | 0.282 |  |
| **Site: Dajea** | 12.642 | 1107.898 | 0.991 |  |
| **Site: Ermei** | 0.646 | 0.612 | 0.292 |  |
| **Site: Ettseng** | -1.341 | 0.581 | 0.021 | * |
| **Site: Jibei** | -17.468 | 1269.043 | 0.989 |  |
| **Site: Jiurua** | 13.821 | 603.185 | 0.982 |  |
| **Site: Lukang** | 0.307 | 0.673 | 0.648 |  |
| **Site: Ponhu** | -0.728 | 0.585 | 0.213 |  |
| **Site: Taipei** | 0.218 | 0.785 | 0.781 |  |
| **Site: Taneia** | -0.808 | 0.591 | 0.171 |  |
| **Site: Tonka** | -0.869 | 1.035 | 0.401 |  |
| **Site: Wujye** | -0.524 | 0.585 | 0.370 |  |
| **Birth HH occupation: craftsman** | -0.317 | 0.455 | 0.486 |  |
| **Birth HH occupation: laborer** | -0.547 | 0.195 | 0.005 | ** |
| **Birth HH occupation: landlord** | -1.295 | 0.818 | 0.113 |  |
| **Birth HH occupation: merchant** | 0.214 | 0.264 | 0.416 |  |
| **Birth HH occupation: unknown** | -0.290 | 0.355 | 0.414 |  |
| **Adopted HH occupation: craftsman** | 1.136 | 1.114 | 0.308 |  |
| **Adopted HH occupation: laborer** | -0.448 | 0.336 | 0.183 |  |
| **Adopted HH occupation: landlord** | -0.286 | 1.286 | 0.824 |  |
| **Adopted HH occupation: merchant** | 0.478 | 0.588 | 0.416 |  |
| **Adopted HH occupation: unknown** | -0.060 | 0.186 | 0.748 |  |

^a^ Sample consists of 1,904 women, with a recorded birth after 1905, adopted to unrelated families of known identities only once and before the age of 10.

^b^ Variable notes are the same as in Table S3
